# Metrology of a Focusing Capillary Using Optical Ptychography

**DOI:** 10.3390/s20226462

**Published:** 2020-11-12

**Authors:** Xiaojing Huang, Evgeny Nazaretski, Weihe Xu, Dean Hidas, Mark Cordier, Benjamin Stripe, Wenbing Yun, Yong S. Chu

**Affiliations:** 1 National Synchrotron Light Source II, Brookhaven National Laboratory, Upton, NY 11973, USA; weihexu@bnl.gov (W.X.); dhidas@bnl.gov (D.H.); ychu@bnl.gov (Y.S.C.); 2 Sigray Inc., Concord, CA 94520, USA; mcordier@sigray.com (M.C.); bstripe@sigray.com (B.S.); wyun@sigray.com (W.Y.)

**Keywords:** X-ray capillary optics, X-ray microscopy, ptychography

## Abstract

The focusing property of an ellipsoidal monocapillary has been characterized using the ptychography method with a 405 nm laser beam. The recovered wavefront gives a 12.5×10.4 μm${}^{2}$ focus. The reconstructed phase profile of the focused beam can be used to estimate the height error of the capillary surface. The obtained height error shows a Gaussian distribution with a standard deviation of 1.3 μm. This approach can be used as a quantitative tool for evaluating the inner functional surfaces of reflective optics, complementary to conventional metrology methods.

## 1. Introduction

Capillaries have been used as one type of reflective X-ray focusing optics for their advantages of high focusing efficiency and generous working distance, compared with peer X-ray optics [1]. They are widely used as the condenser optic for Transmission X-ray Microscopy. Recent fabrication advancement has successfully pushed the focal size to ∼200 nm [2]. Capillaries have been implemented as focusing optics in synchrotron [3] and FEL [4] applications. With the continuous improvements on fabrication processes towards reaching the theoretical focal size around 10 nm [5], capillaries are expected to gain popularity in scanning probe microscope systems.

A capillary collimates X-rays by total external reflection. For a point source or a parallel illumination, the inner surface of a capillary is shaped as a rotational ellipsoidal or parabolic geometry, respectively. The focal spot is located at one of the two focal points of the ellipse or the focus of the parabola. The focusing profile is directly determined by the quality and profile of the reflection surface. As the reflection surface is located at the inner side of the capillary, it is very challenging to use conventional metrology methods to characterize the figure error. On the other hand, the focusing profile can be precisely measured. As an emerging high-resolution microscopy method, ptychography has been used to quantitatively characterize the focusing wavefronts from a wide variety of X-ray optics, including zone plates [6], K-B mirrors [7], compound refractive lens [8], kinoform lens [9], wave guides [10] and multilayer Laue lenses [11]. As the reconstruction gives a quantitative complex-valued wavefront, it can be used to back-calculate the optics aberrations, which provides critical information for improving focusing capability by optimizing fabrication processes or compensating imperfections with aberration-correction optics [12,13].

Here, we report a proof-of-principle work on characterizing the focusing property of an ellipsoidal monocapillary using a 405 nm laser beam. The recovered wavefront gives a 12.5×10.4 μm${}^{2}$ focus. The reconstructed phase profile of the focused beam was used to estimate the height error of the capillary surface, which shows a Gaussian distribution with a standard deviation of 1.3 μm.

## 2. Instrument Setup

**Figure 1:**
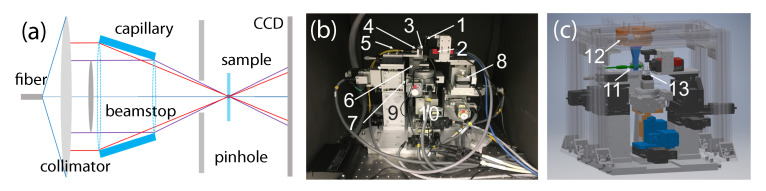
The optical schematic (**a**), photograph (**b**) and Computer Assisted Design (CAD) model (**c**) of the visible light laser-based scanning ptychography microscope installed inside the light-isolating enclosure. The key components are enumerated: 1—surveillance camera; 2—detector Prosilica GT3300 camera; 3—pinhole; 4—collimating laser lens; 5—single mode optical fiber; 6—pinhole manipulation x, y, z; 7—sample rotation; 8—detector manipulation, x, y, z; 9—laser manipulators, x, y; 10—sample linear motions, x, y, z; 11—capillary; 12—6D motion system for capillary alignment; 13—sample mount.

**Figure 2:**
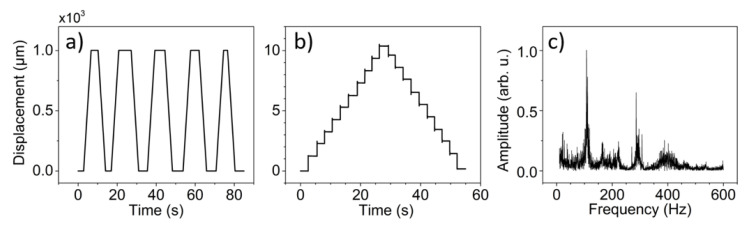
Motion performance of the sample stage. (**a**) Five fly-scan motions over 1 mm range. (**b**) 1 μm step motion over 10 μm range. For both cases motions have been verified using an external laser interferometer mounted on a separate reference structure. (**c**) the X-direction FFT spectrum of the sample stage, fundamental resonances are located above 100 Hz making the system less susceptible to environmental vibrations and cultural noise present in the laboratory.

The developed laser-based ptychography system, in terms of its functionality, is equivalent to a scanning X-ray microscope used for conventional X-ray studies [14]. Figure 1a shows the optical schematic of the microscope system. All key components are labeled in the photograph of the assembled system Figure 1b and the Computer Assisted Design (CAD) model Figure 1c. The developed system is stationed in the R&D labs of the NSLS-II. It is equipped with two interchangeable 10 mW laser diodes (as sources of illumination) operating at the wavelength of 405 nm and 635 nm respectively. Since the wavelength of the laser sources is much larger than that of the X-rays, requirements for scanning ranges, resolutions, and stability are adjusted accordingly. The capillary was mounted on top of a parallel kinematic device (SMARPOD 110.45 from SmarAct) with a 6D manipulation to enable perfect alignment of its optical axis to the incoming laser beam. The control system of the developed laser-equipped microscope is based on the Experimental Physics and Industrial Control System (EPICS), which allows inter-device communication and control over the network by way of the EPICS Channel Access protocol. The Delta Tau Geo Brick, a Linux-based motion control system, is the core of the motion control system. A typical resolution during fly-scanning [15,16,17] does not exceed 100 nm. However, scan areas cover mm-range areas. Figure 2a shows a ramp scan over 1 mm distance while Figure 2b demonstrates a step scan of 10 μm with a 1 μm increment. All scans, their ranges, and resolutions have been verified using external laser interferometry. Figure 2c demonstrates the FFT spectrum of the scanning stage, all fundamental resonance frequencies are located above 100 Hz making the system less susceptible to the background and environmental noises.

## 3. Results

**Figure 3:**
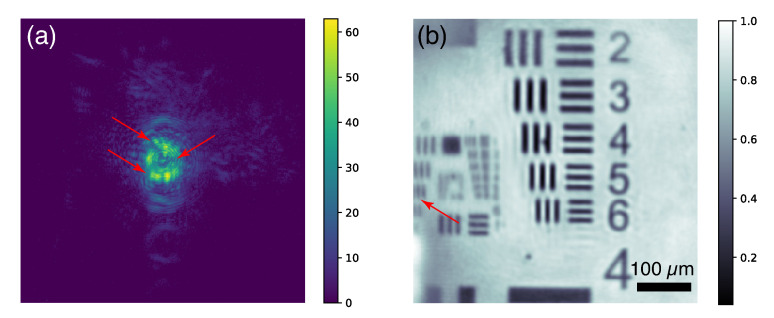
(**a**) A typical data frame of scattering amplitude with 0.02 s exposure time. (**b**) The reconstructed amplitude around the Group 5 features on the resolution chart.

The parameters of the ellipsoidal monocapillary is summarized in Table 1. The position and tip/tilt angles of the capillary were aligned with the fixed laser illumination by manipulating the parallel kinematic device to achieve a symmetric central disk observed on the CCD detector with the strongest intensity and with no boundary interrupted by the capillary edges. A 1951 USAF resolution test chart was placed in the vicinity of the focal plane as the sample during this experiment. The scan trajectory followed a Fermat spiral pattern [18] covering a 200×200 μm${}^{2}$ area with 5 μm radial increments. Such a scan consists of 512 data frames. The scattering intensities were collected by a Prosilica GT3300 CCD camera (Allied Vision, Exton, PA, USA) with a 40% quantum efficiency for 405 nm wavelength and 5.5×5.5 μm${}^{2}$ pixels placed at 19 mm downstream from the sample. The collected data frames were subtracted by a dark background collected at the same condition with the laser diode turned off. Figure 3a shows a typical background-subtracted data frame of a scattering amplitude with 0.02 s exposure time, which gives an average of 33 counts per pixel inside the cropped area. The donut-shaped profile near the scattering center represents the diverging cone of the capillary numerical aperture with a central beamstop. One can also see 3 groups of radial perturbations (pointed by red arrows in Figure 3a) introduced by the beamstop supporting struts. For each data frame, a 300×300 array was cropped and fed to the ptychography reconstruction engine. The reconstruction was performed with the Difference Map algorithm [19] implemented with multiple GPU acceleration [20]. It took 31 seconds to run 500 iterations. The pixel size of the reconstructed image is 2.3 μm. Figure 3b shows the recovered amplitude around Group 5 on the resolution chart. The vertical lines of Element 4 on Group 6 (pointed by the red arrow in Figure 3b) are resolved. The line width in this element group is ∼5.5 μm, which suggests the resolution of the reconstructed image is about this level.

**Figure 4:**
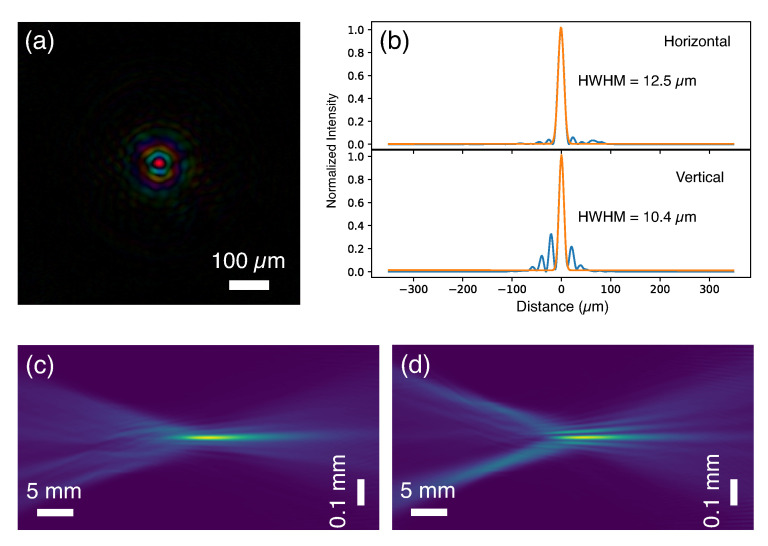
The focused beam produced by the capillary. (**a**) The reconstructed probe function at the focal plane. (**b**) The line profiles of the probe intensity give 12.5 μm and 10.4 μm focal sizes at the horizontal and vertical directions, respectively. The orange curves are Gaussian fit to the intensity profiles. (**c**) and (**d**) are the propagation series over a 50 mm distance in the horizontal and vertical planes, respectively.

The redundant information encoded in overlapped measurements in ptychography makes it possible to reconstruct the complex wavefront of the beam profile together with the object function. To confirm the reconstruction reliability, a second dataset with the same scanning parameters was taken at an adjacent sample area, and both datasets gave almost identical reconstructed probe functions with a correlation coefficient of 99%. Figure 4a shows the recovered focused beam profile with amplitude and phase displayed in brightness and hue, respectively. An intense main peak is well-defined, and it is surrounded by several concentric side rings. The line plots across the main peak in the horizontal and vertical directions were fit with Gaussian functions and gave a 12.5×10.4 μm${}^{2}$ focal size, as shown in Figure 4b. Stronger side lobes can be observed in the vertical direction. The relative intensity of the strongest side lobe is about 30% of the main peak, which is introduced by optics aberrations. The obtained probe was propagated over a 50 mm range using the Angular Spectrum Method [21]. Figure 4c,d are the propagation profiles in the horizontal and vertical planes, respectively. The convergent trend can be seen in both planes with stronger side streaks in the vertical plane.

**Figure 5:**
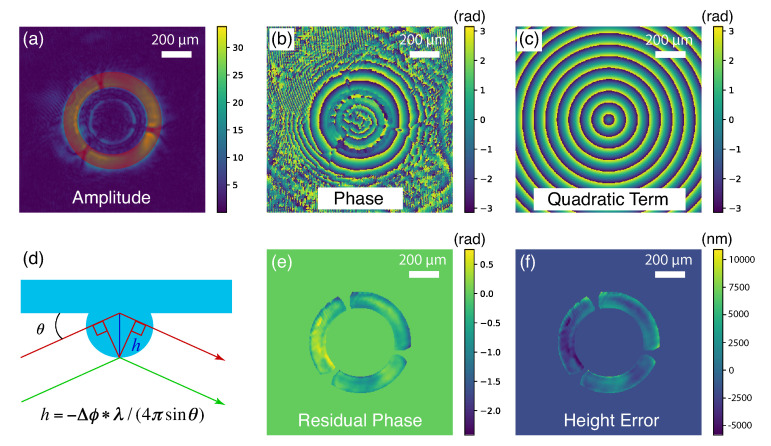
The probe wavefront back-propagated to the capillary exit-plane. (**a**) The back-propagated amplitude map at the capillary exit-plane. The image of the beamstop with three supporting struts are clearly resolved. The opening region is cropped as a mask for further analysis. (**b**) The back-propagated phase map at the capillary exit-plane overlays a circular quadratic phase pattern with characteristic phase feature determined by the capillary quality. (**c**) The ideal quadratic phase pattern for the wavefront propagation. (**d**) A localized height error introduces an extra optical path for the reflected beam deviating from the reflection from the perfect ellipsoidal surface. This extra optical path is represented as an additional phase term in the obtained wavefront. (**e**) The residual phase features obtained by subtracted the ideal quadratic phase pattern (**c**) from the back-propagated phase map (**b**). (**f**) The obtained map of height error converted from the residual phase map using a geometric relation illustrated in (**d**).

The reconstructed complex-valued wavefront carries the quantitative information delivered from the focusing capillary, thus the features in the wavefront can be traced back and related to the artifacts on the reflection surface of the capillary. We firstly propagated the recovered probe wavefront to the exit pupil plane of the capillary using a Fresnel propagator [21]. Figure 5a shows the amplitude map on this plane, where a donut-shaped bright ring with a dark disc is shown in the center with well-defined boundaries. Given the reflection geometry of the capillary, the dark disc and the peripheral area in Figure 5a actually represent the area outside the entrance aperture and the central area blocked by the beamstop, respectively. The three spokes for the beamtop support struts can be sharply seen in this plane as well. Since the beamstop and its supporting struts are not transparent to 405 nm visible light, the incident illumination is completely blocked within these areas. We noticed that there was ∼10% of the intensity outside the exit pupil. In the ideal case, when the collimated beam only reflects once by the inner surface of the capillary and bounces towards the focus, there should be no photons outside the exit pupil. However, the doublet collimator used in this experiment has a 0.009${}^{\circ }$ full-angle divergence. This imperfection in the collimation as well as the scattering from the surface roughness introduced by the height errors are expected to deviate the directions of a portion of photons, which can be bounced multiple times inside the capillary and thus exit the capillary outside the donut-shaped pupil. We defined a mask using the amplitude map on this propagated exit pupil plane to represent the acceptation aperture to the incident illumination, shown as the red ring in Figure 5a.

Figure 5b shows the propagated phase map at the exit pupil plane of the capillary. One can see phase features overlaid on concentric rings of quadratic phase terms created from the wavefront propagation. Since it is exactly known how far the wavefront has been propagated to reach the exit pupil plane, one can calculate the corresponding quadratic phase terms (as shown in Figure 5c), and subtract them from the propagated phase map. The residual phase map is shown in Figure 5e after applying the acceptance mask. For a perfectly fabricated capillary, a uniform residual phase is expected, while for the capillary measured in this work, we observed the residual phase varying within a range of π radian. As the interaction between incident photons and the capillary inner surface is a total reflection, the residual phase can be modeled as the phase shift introduced by the optical path difference of beams reflected from a perfect ellipsoidal surface and a surface with local height errors [2]. As shown in Figure 5d, the relationship between the residual phase Δϕ and the height error *h* can be expressed as h=−Δϕ*λ/(4πsinθ), where λ is the wavelength and θ is the incident angle on the ellipsoidal capillary surface [22]. The converted height error map is shown in Figure 5f.

**Figure 6:**
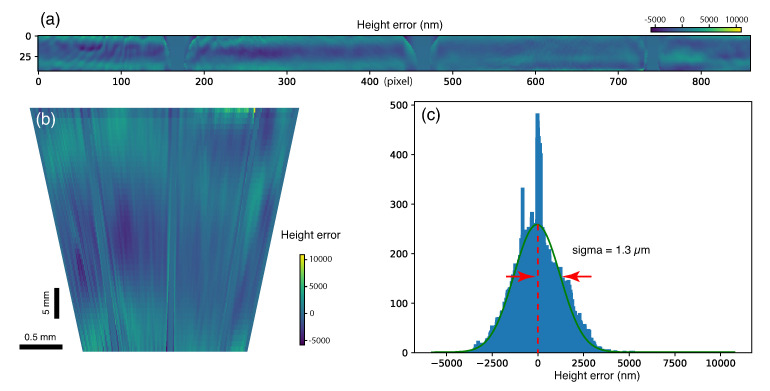
The height error of the capillary surface retracted from reconstructed phases. (**a**) The flattened height error map processed from Figure 5f. (**b**) The height error map interpolated on the capillary inner surface. (**c**) The histogram of the height error shows a Gaussian distribution with a standard deviation of 1.3 μm.

For a better illustration, the height error resides inside the donut-shaped acceptance mask has been mapped to the ellipsoidal inner surface of the capillary. The height error map was firstly flattened to a band (Figure 6a), and then mapped to the capillary surface through interpolation. The obtained height error map is displayed as a fan shape in Figure 6b. As all the usable phase information was encoded in the donut-shaped area at the propagated pupil plane, the resolution along the longitudinal direction was not ideal in this measurement. There are only 45 pixels across the 40 mm long capillary. Nevertheless, the obtained height error shows a relatively slow variation along the longitudinal direction compared with the sagittal direction. The histogram of the overall height errors shows a Gaussian distribution with a sigma of 1.3 μm, as seen in Figure 6c.

## 4. Discussions

To provide a clearly observable figure error in this proof-of-principle laser ptychography measurement, a capillary with a higher-than-usual figure error has been used, instead of a state-of-the-art capillary that can be fabricated at Sigray [23].

The profile of the optical surface along two orthogonal directions was measured at Sigray, which provides additional information about the optical surface [23,24]. After removing a fitted elliptical curve, the obtained height error show a similar wavy profile as we observed along the longitudinal direction in this study, but gives a standard deviation smaller than the number extracted from the ptychography reconstruction. One possible reason is that the metrology measurement at Sigray only samples along the longitudinal direction, while the ptychography measurement gives information on the entire surface but the longitudinal resolution is very poor. For typical capillary optics, the height error in the sagittal direction is expected to be more significant compared with the longitudinal direction, because the sagittal curvature is much smaller and it is much more challenging to control during the fabrication process. Another reason is that the overall height error trend in the orthogonal line measurement can be fitted to an elliptical profile, which slightly differs from the designed parameters. This long-range deviation cannot be removed in the height conversion process from the reconstructed phase in the ptychography measurement, which contributes to the broadening of the distribution of height errors.

The laser diode used in this experiment has a center wavelength λ of 405 nm, with a 20 nm bandwidth Δλ. The longitudinal coherence length can be calculated from $\lambda^{2}/\Delta \lambda =8.2$ μm. Considering the residual phase introduced by the height error varies within a range of ∼π (as shown in Figure 5e), the corresponding optical path difference is expected to around 202.5 nm level, which is significantly smaller than the longitudinal coherence length. Another potential impact arises from how perpendicular the sample surface was relative to the incident illumination. As the scan used in the experiment covered a 200×200 μm${}^{2}$ area, to ensure the surface position difference along the beam propagation direction over the scanned area less than 8.2 μm, the misalignment angle of the sample surface should be smaller than 8.2 μm/200 μm = 0.041 rad = 2.3${}^{\circ }$. The fabrication and assembly accuracy of the sample stage is expected to be better than 0.5${}^{\circ }$. As a result, the longitudinal coherence length is not expected to make a significant impact on this measurement.

The laser beam from the diode was guided out by a fiber with a 2.4 μm diameter. The exit side of the fiber was coupled in the focal plane of a doublet collimator with 33.9 mm focal length, which converted the laser beam to a parallel illumination with ∼5.3 mm diameter. This arrangement can be modeled as a plane 33.9 mm (*z*) away from a 2.4 μm (*d*) pinhole, the corresponding transverse coherence length is ∼λz/d=5.7 mm, which is significantly larger than the entrance aperture of the capillary 1.05 mm. Thus, the transverse coherence property is expected to be sufficiently good for the measurement in this study. This conclusion is supported by the results from a reconstruction with 5 illumination models, where the primary mode contributes to over 88% of the total intensity.

The current laser-based measurement suffers from very limited sampling points along the longitudinal direction, since all the information is packed in a donut-shaped window. One way to ameliorate this limitation is to collect higher spatial frequency signal, thus achieving finer reconstruction pixel size. For the CCD camera used in this experiment, we noticed that the dark noise level was quite high, which shrinks the usable dynamic range for photon detection. In future measurements, accumulating repeated exposures at each scan position can effectively improve the detection dynamic range for achieving higher resolution and increasing pixel numbers representing the longitudinal direction. Similar measurements can be conducted with X-rays at synchrotron facilities, and the achievable spatial resolution will be significantly improved. A few instrumentation aspects may need to be considered for such an experiment. For instance, the focal length of the capillary optics is typically much longer than conventional X-ray lenses, such as zone plates and multilayer Laue lenses [25]; the focal size of the capillary used in this experiment is 2 to 3 orders of magnitude larger than nano-focused X-ray beams [11], thus the sampling condition will be very different, which may require the detector placement significantly further downstream.

In summary, we have built a visible-light laser-based scanning microscope dedicated to ptychography measurements. This setup has been used to characterize the focusing behavior of an ellipsoid monocapillary, which yields a 12.5×10.4 μm${}^{2}$ focus. The reconstructed wavefront has been processed to estimate the height error on the inner reflective surface of the capillary. Slightly different variation trends were observed along the longitudinal and the sagittal directions. The standard deviation of the overall height error of 1.3 μm has been obtained for the measured capillary. The reported characterization approach represents a convenient and quantitative way to map the roughness of the inner functional surface of reflective optics, which can be used as a useful complementary tool alternative to conventional metrology measurements.

## Figures and Tables

**Table 1:** The ellipsoidal monocapillary parameters.

Ellipse Semi-major Axis	2500 mm
Ellipse Semi-minor Axis	2.3 mm
Entrance Diameter	1.05 mm
Exit Diameter	0.64 mm
Mirror Length	40 mm
Beamstop Diameter	0.67 mm

## Abbreviations

The following abbreviations are used in this manuscript:

K-B mirrors	Kirkpatrick-Baez mirrors
FEL	free electron laser
NSLS-II	National Synchrotron Light Source II
GPU	graphics processing unit
CCD	charge-coupled device
